# Beneficial non-anticoagulant mechanisms underlying heparin treatment of COVID-19 patients

**DOI:** 10.1016/j.ebiom.2020.102969

**Published:** 2020-08-25

**Authors:** Baranca Buijsers, Cansu Yanginlar, Marissa L. Maciej-Hulme, Quirijn de Mast, Johan van der Vlag

**Affiliations:** a Department of Nephrology, Radboud Institute for Molecular Life Sciences, Radboud university medical center, Nijmegen, The Netherlands; b Department of Internal Medicine, Radboud university medical center, Nijmegen, The Netherlands

**Keywords:** COVID-19, Heparin, Low molecular weight heparin, Heparanase, Inflammation, COVID-19, Coronavirus disease-2019, LMWH, low molecular weight heparin, SARS-CoV-2, severe acute respiratory syndrome coronavirus 2, SARS-CoV, severe acute respiratory syndrome coronavirus, MERS-CoV, Middle East respiratory syndrome coronavirus, ARDS, acute respiratory distress syndrome, AKI, acute kidney injury, ICU, intensive care unit, GAGs, glycosaminoglycans, IU, international unit, HPSE, heparanase, HS, heparan sulphate, SGP, spike glycoprotein, NETs, neutrophil extracellular traps

## Abstract

Coronavirus disease-2019 (COVID-19) is associated with severe inflammation in mainly the lung, and kidney. Reports suggest a beneficial effect of the use of heparin/low molecular weight heparin (LMWH) on mortality in COVID-19. In part, this beneficial effect could be explained by the anticoagulant properties of heparin/LMWH. Here, we summarise potential beneficial, non-anticoagulant mechanisms underlying treatment of COVID-19 patients with heparin/LMWH, which include: (i) Inhibition of heparanase activity, responsible for endothelial leakage; (ii) Neutralisation of chemokines, and cytokines; (iii) Interference with leukocyte trafficking; (iv) Reducing viral cellular entry, and (v) Neutralisation of extracellular cytotoxic histones. Considering the multiple inflammatory and pathogenic mechanisms targeted by heparin/LMWH, it is warranted to conduct clinical studies that evaluate therapeutic doses of heparin/LMWH in COVID-19 patients. In addition, identification of specific heparin-derived sequences that are functional in targeting non-anticoagulant mechanisms may have even higher therapeutic potential for COVID-19 patients, and patients suffering from other inflammatory diseases.

## 1 Introduction

Coronavirus disease-2019 (COVID-19) is caused by the severe acute respiratory syndrome coronavirus 2 (SARS-CoV-2). SARS-CoV-2 is closely related to other coronaviruses that emerged in the last two decades including the severe acute respiratory syndrome coronavirus (SARS-CoV), and the Middle East respiratory syndrome coronavirus (MERS-CoV). Compared to SARS-CoV, and MERS-CoV, SARS-CoV-2 appears to spread more efficiently within the human population and officially caused a pandemic by mid-March 2020 [1].

COVID-19 primarily presents with common flu symptoms such as fever, muscle pain, and cough, and in severe cases causes acute respiratory distress syndrome (ARDS) [2]. Besides ARDS, severely ill COVID-19 patients may progress to multi-organ dysfunction. Another common complication of COVID-19 is acute kidney injury (AKI), and proteinuria, which is leakage of protein into the urine. It is reported that around 40% of COVID-19 patients developed proteinuria upon hospital admission [3]. Moreover, in the intensive care unit (ICU) setting 90% of patients presented with AKI [3]. Thus AKI is considered a negative prognostic factor regarding survival of COVID-19 patients [4]. Similar to pulmonary oedema, which causes ARDS, proteinuria is caused by a compromised endothelial glycocalyx, which is normally comprised of a thick layer of negatively charged glycosaminoglycans (GAGs). The integrity of the endothelial glycocalyx is crucial for endothelial barrier function, especially in the lungs [5,6], and in the kidneys [7,8], and this endothelial barrier seems to be disrupted in COVID-19 patients. Finally, autopsies from COVID-19 patients identified the presence of thrombus formation in the microvasculature, which suggests that coagulation is an important contributor in organ failure of COVID-19 patients [9,10].

**Fig. 1:**
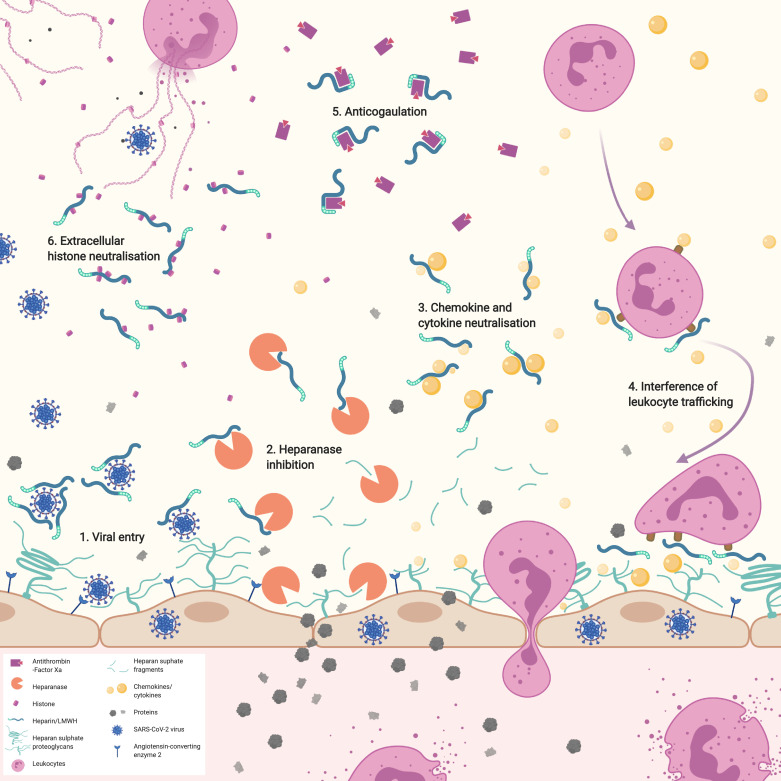
Summary of the potential beneficial mechanisms of heparin/low molecular weight heparin (LMWH) underlying treatment of COVID-19 patients. 1. Reducing viral entry. Heparan sulphate, and heparin/LMWH have been shown to interact with SARS-CoV-2 spike glycoprotein. 2. Inhibition of heparanase activity. Heparin/LMWH has been shown to inhibit heparanase activity, which is increased in COVID-19 and associated with disease severity. 3. Neutralisation of the biological effect of chemokines, and cytokines. Heparin/LMWH interact with chemokines, and cytokines, including those produced in the ‘cytokine storm’ in COVID-19. 4. Interference with leukocyte trafficking. Heparin/LMWH neutralisation of chemokine, and cytokines may impact on leukocyte recruitment and trafficking to sites of inflammation, either via neutralisation of chemokine, and cytokines or through direct interaction with leukocyte cell surface ligands, i.e. selectins, and integrins, to prevent leukocyte attachment, and extravasation. 5. Anticoagulation. Heparin/LMWH promotes anticoagulation via anti-thrombin III binding. 6. Neutralisation of extracellular cytotoxic histones. Heparin/LMWH act as a neutralising compound for histones via ionic interactions of the negatively charged chemical groups with the positively charged extracellular histones released during COVID-19.

There are no COVID-19-specific treatments or vaccines available, and care is primarily supportive. The broad spectrum antiviral, Remdesivir, shows promise by inhibiting viral replication of SARS-CoV-2 in animal models and shortening the time for clinical improvement [11], as well as the corticosteroid dexamethasone, which showed a reduction in 28-day mortality in patients requiring oxygen therapy, or mechanical ventilation upon administration, as described in a recent preliminary report [12]. As cohort studies suggest a high rate of thromboembolic complications among hospitalised patients [13], prophylactic administration with LMWH for hospitalised COVID-19 patients is recommended [14], whereas some experts recommend higher doses for critically ill patients. LMWH is preferred over unfractionated heparin due to the decreased risk of bleeding, good predictability, dose-dependent plasma levels and longer half-life [15,16]. Data from retrospective studies suggest that the use of heparin/LMWH may improve outcome in COVID-19, although evidence of prospective trials is needed before more firm conclusions can be drawn [17], [18], [19]. Systemic anticoagulation was associated with improved in-hospital survival among hospitalized patients with COVID-19, but only in the subgroup of intubated patients [19]. Notably, a retrospective study from China found systemic anticoagulation mainly with LMWH to be associated with a lower mortality, however only 15% of patients in this cohort were using LMWH and the survival benefit was restricted to those with a high sepsis-induced anticoagulant score, or D-dimer level [17]. In addition to functioning as anticoagulants, heparins have other therapeutic functions that are relevant for the treatment of COVID-19-associated clinical manifestations, i.e. neutralisation of inflammatory chemokines, and cytokines, such as CXCL-1, IL-6, and IL-8 that play a key role in ARDS; neutralisation of extracellular cytotoxic histones and by interfering with leukocyte trafficking [20]. Since the biological roles of heparins are versatile, it is currently debated via which mechanisms heparin/LMWH could be beneficial for COVID-19 patients (Fig. 1) [20].

One important, currently overlooked characteristic of heparin/LMWH in the pathogenesis of COVID-19 is the inhibitory effect on heparanase (HPSE) [21]. HPSE mediates the disruption of the endothelial barrier, in particular by degrading the endothelial glycocalyx, which has also been described for several pathologies such as pulmonary oedema, and proteinuric kidney disease [5,7,8,[22], [23], [24], [25]]. This suggests that increased HPSE activity may play a role in the severe clinical manifestations of COVID-19, including ARDS, and AKI. Notably, in a recent preprint we showed increased plasma HPSE activity in COVID-19 patients [26].

The aim of this review is to summarise the literature concerning the non-anticoagulant functions of heparin/LMWH with a special focus on: the inhibition of HPSE activity, neutralisation of chemokines/cytokines, inhibition of leukocyte trafficking, and neutralisation of extracellular cytotoxic histones in the circulation. We propose that therapeutic treatment with heparin/LMWH will interfere with several pathological processes in COVID-19 patients, thereby increasing their survival rate.

## 2 Potential beneficial non-anticoagulant effects of heparin/LMWH treatment for COVID-19 patients

### 2.1 Heparin/LMWH inhibits HPSE activity

As outlined, leakage of proteins, and fluid across the endothelial barrier is manifested in most severe clinical outcomes of COVID-19, including ARDS, and proteinuria. In normal physiological conditions, the endothelial glycocalyx prevents leakage of proteins in a charge and size-dependent manner [8]. Heparan sulphate (HS) is the most abundant sulphated GAG in the glycocalyx and is the main contributor to its negative charge-dependent barrier function [8,22]. HPSE is the only known mammalian enzyme capable of degrading HS. Hence, prolonged, increased activity of HPSE compromises the glycocalyx, which causes a subsequent loss of endothelial barrier function and leaky blood vessels that contribute to lung and kidney complications, as observed in ARDS and proteinuric kidney diseases [5,7,8,[22], [23], [24], [25]]. HPSE also plays a predominant role in systemic vascular leakage induced by sepsis [25,27], or severe dengue disease [28]. Attenuation of HPSE activity in models of aforementioned diseases prevented endothelial hyperpermeability, and protein leakage [25,[27], [28], [29], [30]], thereby protecting from severe injury and preserving lung, and kidney function [25,31]. Therefore, the loss of endothelial barrier function in COVID-19, leading to pulmonary oedema, and proteinuria, may be in part the result of increased HPSE-mediated degradation of the glycocalyx. Consequently, inhibition of HPSE activity may benefit COVID-19 patients with complications caused by a leaky vasculature, i.e. ARDS, proteinuria, by preventing glycocalyx dysfunction. Importantly, heparin/LMWH, and chemical heparin derivatives have proven to be potent HPSE inhibitors [21,[32], [33], [34]]. In line with this, in a recent preprint we showed that non-ICU patients receiving the LMWH dalteparin in a prophylactic dose of 5000 IU daily had a significantly lower HPSE activity, suggesting that clinically relevant inhibition of HPSE activity can already be reached using prophylactic-dose LMWH [26]. Taken together, we hypothesise that inhibition of increased HPSE activity by heparin/LMWH could be one of the main mechanisms to reduce severe clinical manifestations of COVID-19.

### 2.2 Heparin/LMWH has anti-inflammatory properties

Heparin/LMWH possesses multiple anti-inflammatory properties and various mechanisms underlying the anti-inflammatory effect of heparin/LMWH have been proposed [16,35,36]. The potential of heparin/LMWH as a therapeutic compound for inflammatory diseases has been supported by clinical trials [36], experimental models in bronchial asthma, ulcerative colitis, burns, ischemia-reperfusion, arthritis, and peritonitis [35,37]. In addition to its contribution to endothelial barrier function, the endothelial glycocalyx also mediates several inflammatory processes. The specific sulphation patterns of GAGs observed in a healthy endothelial glycocalyx attenuates binding of chemokines and leukocytes to the endothelial cell surface [38]. However, the structure of GAGs changes under inflammatory conditions, which facilitates the binding of chemokines, as well as selectins, and integrins expressed by leukocytes [5,39]. Increased HPSE activity is also involved in the development of a proinflammatory glycocalyx. Cells exposed to HPSE show an enhanced response to stimuli, such as proinflammatory cytokines [40,41]. Furthermore, HS fragments shed by HPSE contribute to the inflammatory extracellular milieu, for example, by the release of sequestered chemokines, and/or the binding of HS fragments to toll-like receptors [42,43]. Importantly, HPSE deficiency and/or HPSE inhibition supports beneficial outcomes in experimental inflammatory lung and kidney disease, which may be also relevant for the clinical complications of COVID-19 [22,25,31,44,45].

COVID-19 is associated with production of high levels of pro-inflammatory cytokines [46]. Heparin/LMWH are able to bind to the vast majority of chemokines and cytokines including IL-8 [47,48]. Heparin/LMWH binding of chemokines/cytokines may neutralise their biological effect. Under inflammatory conditions, chemokine and cytokine binding to endothelial cell surface GAGs promotes activation and trafficking of leukocytes, i.e. endothelial cell-bound IL-8 mediates chemotaxis of neutrophils. However, exogenous heparin and LMWH disrupts this process by competing with endothelial cell surface HS for IL-8 [47,48]. Besides neutralisation of chemokine and cytokine function, both heparin and LMWH has also been shown to inhibit cytokine synthesis including TNF-α, IFN-γ, IL-6, and IL-8 via inhibition of NF-κB signalling [16,49]. In addition, LMWH may also interfere with the bradykinin pathway, which has been proposed in a recent preprint to be over-activated in COVID-19 due to the consumption of angiotensin converting enzyme-2 during viral entry [50]. Endothelial cell surface GAGs regulate activation of bradykinin pathways and HS degradation by HPSE promotes proteolytic bradykinin generation from high molecular weight kininogen [51]. Therefore, LMWH may inhibit bradykinin formation both via inhibition of HPSE activity and by its ability to bind high molecular weight kininogen, thereby attenuating the local inflammation and vascular leakage in COVID-19 [52].

COVID-19 is also associated with the influx of immune cells [53,54]. Notably, leukocyte rolling, firm adhesion to endothelial cells, and transmigration can be attenuated by heparin/LMWH [55,56]. Several studies demonstrated that heparin/LMWH interferes with leukocyte rolling, adhesion, and migration, via binding to L-selectin, P-selectin, and Mac-1/CD11b expressed by leukocytes, consequently competing with the interaction of endogenous binding sites present in endothelial cell surface GAGs [57,58]. Since leukocyte activation and trafficking play a central role in the inflammatory response of COVID-19 [53,54], inhibition of leukocyte adhesion and migration by heparin/LMWH could dampen the immune response.

Finally, heparin/LMWH inhibits complement activation [16,59]. The complement system plays an important role in innate immune defence and shaping of adaptive immune responses. Activation of the complement system leads to many processes such as opsonisation and phagocytosis of pathogens, chemotaxis of neutrophils, and release of inflammatory mediators. Excessive complement activation has been proposed to contribute to systemic thrombosis in COVID-19 patients [60,61]. Complement inhibition in SARS-CoV [62], and MERS-CoV [63] murine models was associated with favourable outcomes, suggesting that the same may be true for COVID-19. Heparin/LMWH interferes with complement activation at multiple levels, including binding to C1q and inhibiting the cleavage of C2, C3, and C4 [16]. Heparin/LMWH therefore inhibits activation of all three of the complement pathways at various points.

In summary, the most severe complications of COVID-19 involve inflammation, including neutrophil infiltration [53], of the lung, and kidney. The anti-inflammatory properties of heparin/LMWH may therefore facilitate dampening of the inflammatory response via multiple mechanisms.

### 2.3 Heparin/LMWH reduces viral entry to host cells

Many viruses (including some coronaviruses) utilise cellular HS as co-receptors for cell attachment [64,65], enabling a localised increase in viral particle concentration to maximise infection rates [66]. Similarly, a novel GAG-binding motif was identified within the SARS-CoV-2 spike glycoprotein (SGP), the viral fusion protein responsible for receptor binding and fusion of the viral and host membranes, which is not present in the SGPs of SARS-CoV, or MERS-CoV [67]. Moreover, it was shown in a preprint that SARS-CoV-2 SGP binds more tightly to immobilised heparin than the SARS-CoV, and MERS-CoV SGPs [68]. Recent preprints by using competitive binding approach showed that both soluble heparin, highly sulphated HS and heparin-derived tetrasaccharides inhibited binding of SARS-CoV-2 to immobilised heparin [68], [69], [70]. SARS-CoV-2 entry into Vero cells *in vitro* was also competitively inhibited by heparin [71], illustrating a functional role of this newly identified SARS-CoV-2 SGP GAG-binding motif for viral host infection. Using a high titre lentivirus pseudotyped with SARS-CoV-2 SGP, a separate study then showed that unfractionated heparin (IC_50_, 599 ng/L) and LMWH (enoxaparin, IC_50_, 108 µg/L) both effectively inhibited infection of HEK293T cells [66]. This potency in inhibition of SARS-CoV-2 SGP/GAG binding mechanism suggests that heparin/LMWH may have immediate therapeutic potential by preventing SARS-CoV-2 infection, thereby attenuating disease severity in COVID-19 patients.

### 2.4 Heparin/LMWH neutralises circulating histones

SARS-CoV-2 has been suggested to induce different forms of cell death including apoptosis [72], and neutrophil extracellular trap (NET) formation [53,73]. Excessive endothelial cell death could directly cause disruption of the endothelial barrier leading to vascular leakage of proteins and fluid. Notably, NETs have been shown to induce endothelial-to-mesenchymal transformation *in vitro* and protein leakage across endothelial monolayers [74,75]. In particular, NOX-independent NETs activated endothelial cells, which was accompanied by diminished barrier function [76].

Histones are highly conserved positively charged proteins, which are essential for chromatin structure and regulation of gene expression. However, when present in the extracellular space upon cell death, histones induce an inflammatory response and are highly cytotoxic contributing to necrosis, apoptosis and the formation of NETs [77,78]. Recent studies have reported that NETs are present in tissues and the circulation of COVID-19 patients [75], suggesting that their cytotoxicity contributes to disease. Negatively charged heparin and, desulphated heparin oligosaccharides has been demonstrated to neutralise the cytotoxic effect of positively charged histones, thereby potentially reducing organ damage [79], [80], [81]. In summary, heparin/LMWH may neutralise the inflammatory and cytotoxic effects of extracellular histones in COVID-19.

## 3 Conclusions, outstanding questions and future perspectives

Due to the multiple inflammatory and pathogenic mechanisms targeted by heparin/LMWH, it is warranted to conduct clinical studies that evaluate therapeutic doses of these compounds in COVID-19 patients. Although a prophylactic dose of LMWH is associated with a reduced HPSE activity in non-ICU COVID-19 patients [26], regarding the other possible non-anticoagulant effects, no data is currently available as to whether heparin/LMWH in their usual prophylactic, or therapeutic dosage are effective to prevent viral entry, to neutralize cytokines and histones, and to interfere with leukocyte trafficking. In fact, the optimal anticoagulant dosing in patients with COVID-19 is currently uncertain. Studies suggest a high rate of thromboembolic complications among hospitalized patients with COVID-19, particularly in patients admitted to the ICU, and often despite prophylactic-dose anticoagulation [13]. This has led some experts to recommend a higher-intensity thromboprophylaxis with intermediate or even therapeutic dosages of LMWH in critically ill patients with COVID-19 [13]. The balance between the risks of thrombosis, and the possible beneficial non-coagulant effects of higher LMWH dosing on the one hand, and the risks of bleeding on the other hand await further study. Considering the structural diversity of heparin/LMWH, in the long term well-defined, heparin-derived structures should be identified that interfere with SARS-CoV-2 cellular entry, COVID-19 related HPSE activity, chemokine binding, leukocyte trafficking, and histone neutralisation, in analogy to the heparin-based pentasaccharide (Arixtra/Fondaparinux) that mediates anticoagulation via antithrombin III. A mixture of these well-defined, heparin-derived compounds could be beneficial for the outcome of COVID-19 patients, as well as for patients suffering from other inflammatory diseases.

## 4 Search strategy and selection criteria

Data for this review were identified by searches of PubMed, and preprint servers, and references from relevant articles using the search terms “COVID-19”, “Heparin”, “Non-anticoagulant functions of heparin”, “Low molecular weight heparin”, “ARDS”, “Kidney dysfunction”, “Endothelial barrier dysfunction”, “Heparanase”, “Heparan sulphate”, “Viral entry”, ”Heparanase inhibition”, “Inflammation”, “Complement system”, and “Neutrophil extracellular traps”.

## Author contributions

BB, CY, MLMH, QM, and JvdV contributed to drafting the manuscript. MLMH created Fig. 1. BB, CY, and JV conceived the idea. JvdV initiated and supervised writing of the manuscript, and secured funding.

## Declaration of competing interest

The authors have declared that no conflict of interest exists.
